# Nutritional value and consumer acceptance of food products fortified with edible insects: a systematic review

**DOI:** 10.1080/23311932.2025.2602907

**Published:** 2026-01-02

**Authors:** Sindiswa Zondo, Thinandavha Caswell Munyai, Muthulisi Siwela, Rob Slotow, Zabentungwa Thakasile Hlongwane

**Affiliations:** a School of Agriculture and Science, University of KwaZulu-Natal, Durban, South Africa; b School of Agriculture and Science, University of KwaZulu-Natal, Scottsville, South Africa; c Oppenheimer Fellow in Functional Biodiversity, School of Agriculture and Science, University of KwaZulu-Natal, Scottsville, South Africa

**Keywords:** Malnutrition, edible insects, nutritional composition, insect-fortified food, Nutrition, Food Additives & Ingredients

## Abstract

Food insecurity and malnutrition remain major global challenges, with an estimated 691–783 million people affected worldwide in 2022. FAO promotes edible insects as a sustainable nutrient source to improve food and nutrition security, especially in sub-Saharan Africa where protein, zinc, and iron deficiencies are common. This review evaluated the effect of fortifying popular foods that are nutrient-deficient with edible insects on their nutritional value and consumer acceptance. A systematic search of peer-reviewed literature was conducted using Web of Science, Scopus, and Google Scholar, following PRISMA guidelines. Publications included primary data on the nutritional value of insect-supplemented foods without restrictions on food types or insect species. The search yielded 73 eligible papers listing 27 insect species used in 16 food products. Food products supplemented with edible insect meal contained double the protein, iron, and zinc compared to controls, but carbohydrate content decreased by half. Bakery products were the most supplemented with edible insects. Edible insects improved nutritional value without negatively affecting consumer acceptability. Therefore, incorporating nutrient-rich insects into popular nutrient-deficient foods can help promote their use and address malnutrition.

## Introduction

According to the United Nations Department of Economic and Social Affairs (2021), the global population will reach at least 9.6 billion by 2050, which will require a 70% increase in food production to support the growing population. Population growth will worsen the issues of malnutrition and food insecurity, which are current serious global problems (Khan & Ali, 2023). Although malnutrition is linked with acute and persistent hunger, the underlying cause is a lack of a balanced diet rather than a lack of food (Behrman et al., 2004). For example, obesity and overweight are types of malnutrition (overnutrition) that occur when there is food, but no balanced diet (Kobylińska et al., 2022). Malnutrition (both undernutrition and overnutrition) can cause serious health problems, particularly in pregnant women, lactating women, children, and the elderly (FAO, 2017). In 2022, 149 million children were reported to be stunted, 45 million were wasted, and 37 million were obese globally (WHO, 2022). Protein-energy malnutrition (PEM) remains a major public health concern in developing countries, particularly in Africa and Southern Asia, resulting in morbidity, death, stunted growth, and impaired neurobehavioral development in children (Adegboye, 2022). According to the World Health Organisation (WHO, 2022), undernutrition accounts for around 60% of deaths that occur in children under the age of five years in underdeveloped countries. FAO et al. (2023) reported that in 2022, 691–783 million people worldwide were faced with different levels of hunger. Out of these, 345 million people are faced with acute hunger and starvation, many of whom live in underdeveloped nations, including Southern Asia and sub-Saharan Africa, where malnutrition is prevalent (FAO et al., 2023). These figures are likely to persist if drastic measures to mitigate food and nutrition insecurity are not implemented (FSIN and Global Network Against Food Crises, 2023).

South Africa is faced with a double burden of malnutrition (undernutrition and overnutrition) (Gebremichael et al., 2025). A total of 155 children died of malnutrition in South Africa during the first half of 2025 (Medical Brief, 2025). However, several strategies have been implemented to address malnutrition in South Africa (Bell et al., 2024; Bourassa et al., 2023; Siwela et al., 2020). These strategies include commercial food fortification with micronutrients, supplementation with adequate quality protein, vitamins, and amino acids, and dietary diversification to increase micronutrient-rich food productivity (Siwela et al., 2020; Verma et al., 2024). However, these strategies have been in place for a long time and are not sustainable (Siwela et al., 2020). The above-stated strategies have not adequately addressed malnutrition because, among other factors, supplements and fortified and diversified food are expensive and only reach the population that can afford the products (Awobusuyi et al., 2020; Duan et al., 2023). Additionally, Bourassa et al. (2023) suggested that proper planning and execution need to be implemented in order for these strategies to work properly. Most of the strategies that have been implemented or suggested are either short-term solutions or are only accessible to households that can afford them (Bourassa et al., 2023). Therefore, long-term, sustainable, innovative, and affordable approaches must be implemented to address this challenge.

The anticipated growth in population will result in an increased demand for conventional protein sources, such as meat, fish, and chicken (Borges et al., 2022). On the other hand, there are environmental concerns about the increased production of conventional protein sources, especially animal-source foods—continuous land clearing and high greenhouse gas emissions are some of the major causes of concern (Borges et al., 2022; Henchion et al., 2017; Lynch et al., 2018; Messina et al., 2023; Oonincx & de Boer, 2012). Livestock production requires more land, is also associated with extensive water use, and produces an excessive quantity of greenhouse gases, including carbon dioxide, methane, nitrous oxide, and ammonia, all of which contribute significantly to global warming (Li et al., 2025; Scholtz et al., 2013). In addition, animal-source foods are generally not economically accessible to most communities in developing regions (Khatun et al., 2021). Whereas, relative to animal-sourced foods, edible insects have a much lower negative impact on the environment (Igual et al., 2021).

To mitigate the negative impact of livestock production on the environment, FAO (2017) has advocated for the restricted use of livestock as a source of protein. Plant-based protein alternatives were found to help address protein shortage and limit the use of traditional animal-source foods (Estell et al., 2021). However, their digestibility posed a challenge for humans (Estell et al., 2021). One of the significant issues with plant-based protein is the low digestibility and solubility of plant proteins resulting from the presence of antinutritional compounds (Hadi & Brightwell, 2021). Hence, innovative, environmentally friendly, affordable, and sustainable alternatives are urgently required. Thus, FAO (2017) has recommended edible insects as an acceptable protein alternative as they are affordable, environmentally sustainable, high in protein, and of good nutritional value.

Entomophagy is not a new practice; it has been a part of many people’s diets worldwide for centuries (Olivadese & Dindo, 2023). However, in some parts of sub-Saharan Africa, the practice has declined dramatically over the years due to the adoption of Western diets to the extent that the younger generations, particularly in urban areas, do not know about the consumption of edible insects (Hlongwane et al., 2021). Fear and discomfort associated with consuming insects have also been listed as among the top reasons for the decline in the practice (Akande et al., 2023; Bawa et al., 2020; Hlongwane et al., 2021). Therefore, there is a need to improve consumer acceptability of edible insects and thus promote their utilisation for improved food and nutrition security (Bawa et al., 2020; Duku et al., 2023; Gantner et al., 2022; Van Huis, 2016). This review aims to evaluate the effect of supplementing common food products with edible insect meal on their nutritional value. The objectives of the review were to (1) examine the evidence that supplementing food products with edible insects improves their nutrient content and (2) evaluate published data on consumer perception and acceptance of food products supplemented with edible insects.

## Materials and methods

### Search strategy

The PRISMA guidelines were followed to obtain information about the nutritional value and consumer acceptability of food products fortified with edible insect meal (Takkouche & Norman, 2011). A literature search was conducted using the Thomson Reuters’ Web of Science, Scopus, and Google Scholar databases to identify relevant peer-reviewed publications focused on food products supplemented with edible insect meals across all continents. The following search terms were used: ‘food product enriched with edible insect’, ‘biscuits supplemented with edible insects’, ‘bread supplemented with edible insects’, ‘cookies enriched with edible insects’, ‘ice cream enriched with edible insects’, ‘muffins enriched with edible insect’, ‘porridge enriched with edible insect powder’, ‘cereal products enriched with edible insect powder’, and ‘insect powder food products’. These keywords, along with their synonyms, were combined using Boolean operators (AND, OR & NOT), including combinations such as ‘nutritional composition AND/OR nutritional value’, ‘supplemented AND/OR enriched’. Additionally, we reviewed the references cited in the selected articles to identify any relevant studies that were not captured in our initial search. We also checked the references in the selected research articles that may be relevant to the current study, but did not appear in our search.

### Inclusion and exclusion criteria

We included original research articles focusing on the nutritional value and consumer acceptability of food products enriched with edible insect meals. We included papers that were published before June 2025. There were no restrictions on the type of food products developed with edible insects, the type of insects used, the country of origin of the insects, and the year of publication. Only studies published in English were included. Conference proceedings, editorial material, and technical reports were excluded from the review.

### Data quality

To evaluate the quality of publications included in this systematic review, we screened each publication based on the following criteria: ensuring that all the relevant information, such as the author’s names, publication, year of publication, article title, and Journal information, was clearly stated. We then confirmed that the publications had been peer-reviewed, ensuring that the journals in which the papers were published have a strong reputation in the field and are relevant to the current research topic. Furthermore, we reviewed the study design and methodology to confirm their scientific standing (credibility, rigour, and validity), which included the sample size and measurement methodology. We then checked to see if any potential biases might have affected the outcome of the study, such as funder interests. Lastly, we confirmed that the studies would yield pertinent data for the current review.

## Results

**Figure 1:**
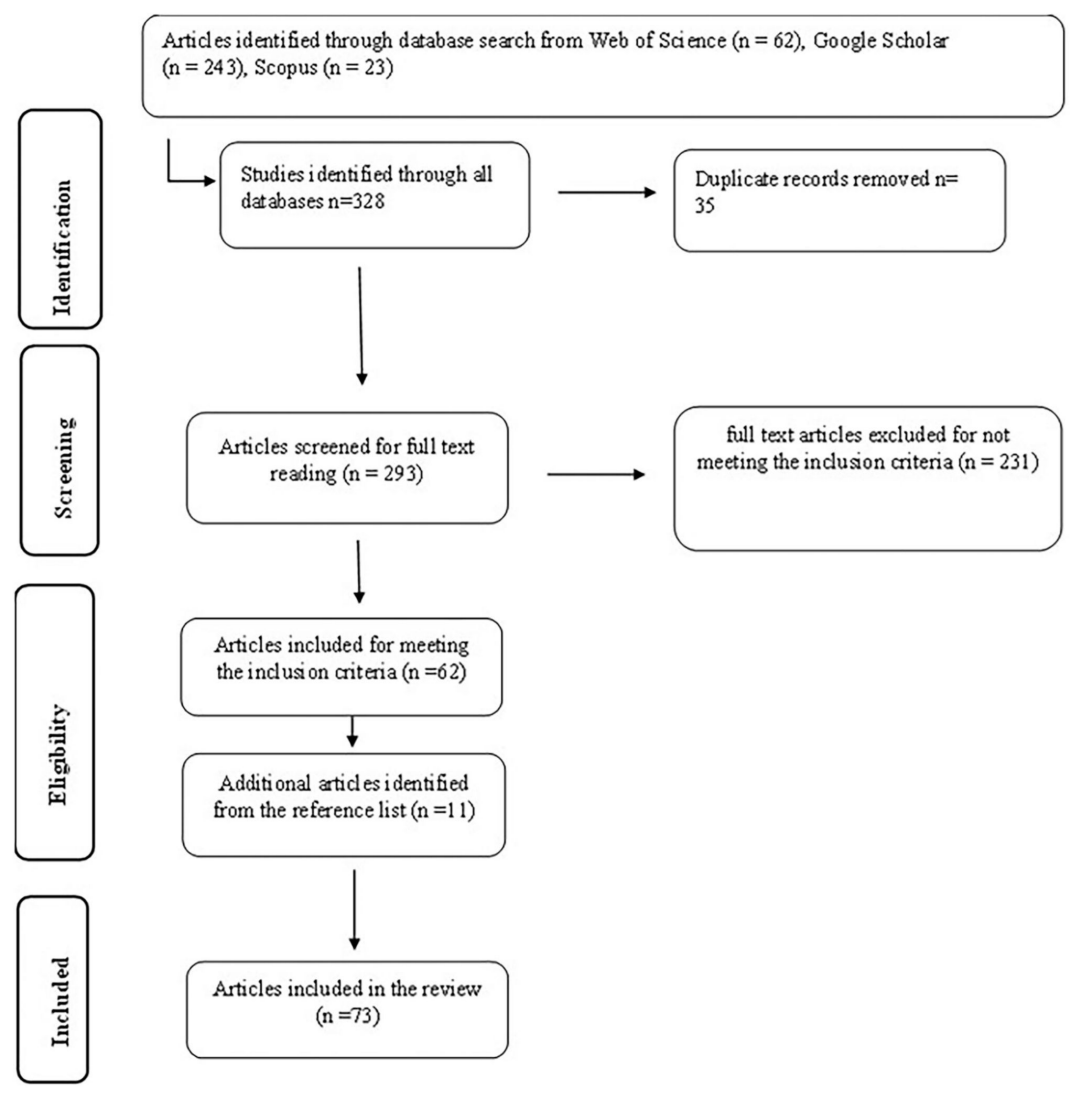
Flow chart of the study selection process for a systematic review of the nutritional value and consumer acceptability of food products fortified with edible insects.

**Figure 2:**
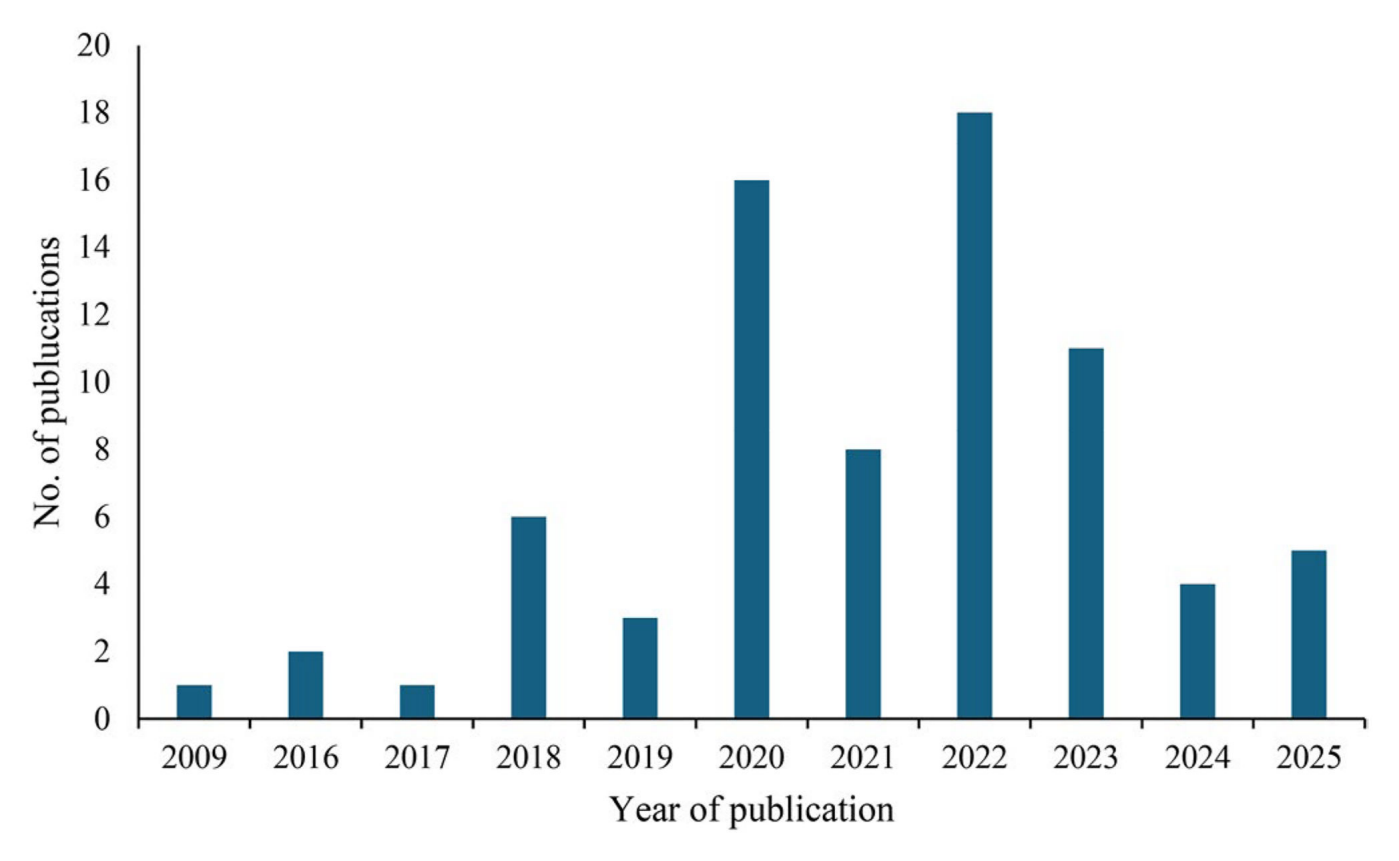
Peer-reviewed articles focusing on the nutritional value and consumer acceptability of food products supplemented with edible insects published over the years.

A total of 328 relevant peer-reviewed articles were identified from the three databases stated above. After reading the title and screening the abstract, 35 duplicates were removed after combining the records from the three databases. After duplicate and abstract screening, 293 articles remained for full-text screening. After full-text screening, 62 articles met the inclusion criteria and were included in the final analysis. An additional 11 articles were retrieved from screening the references. Therefore, a total of 73 articles were included in this review (Figure 1). The highest number of publications was recorded in 2022, followed by 2020, while the lowest number of publications was in 2009 and 2017 (Figure 2). The studies included in the current review were conducted in both developing regions (59%) and developed regions (41%).

**Figure 3:**
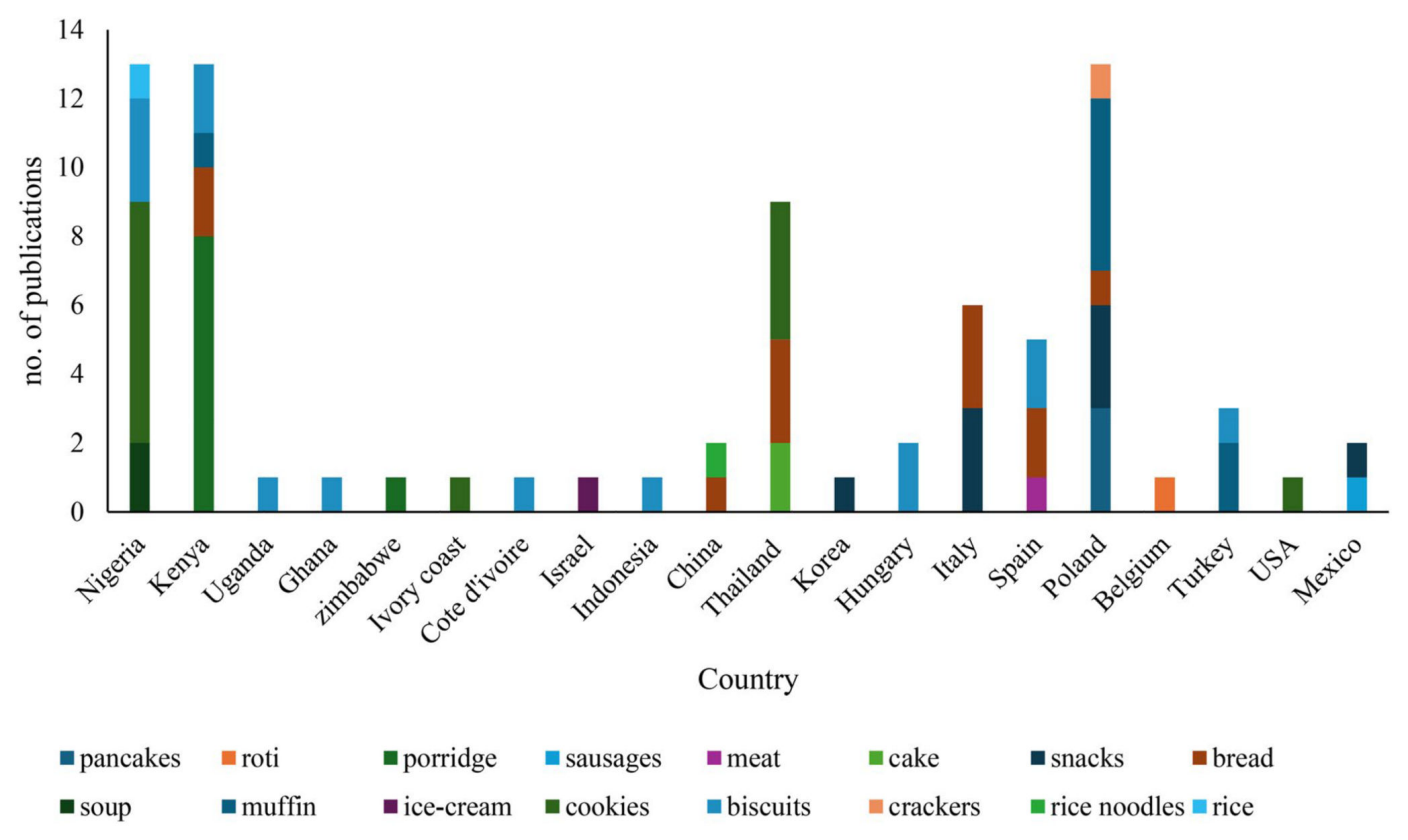
Number of publications per food product fortified with edible insects in different countries.

A total of 16 food products were fortified with edible insects (Figure 3). Poland, Nigeria, and Kenya had the highest number of publications and food products fortified with edible insects, followed by Thailand, while Uganda, Ghana, Zimbabwe, Ivory Coast, Cote d’Ivoire, Israel, Indonesia, Korea, Belgium, and the USA had the lowest number of publications and food products fortified with edible insects (Figure 3). As high as 74% of the reported food products were bakery products, the most used food products in both developing and developed countries, followed by snacks, including protein bars, energy bars, puffed-rice snacks, and nut bars. In contrast, roti and soup were the least used food products in developing and developed countries (Figure 3).

The food products fortified with edible insects exhibited a significant increase in nutrient content compared to the control, with the increase being proportional to the concentration of edible insects (Table 1 and Appendix A). The highest protein content was observed in bread fortified with 90% cricket meal (64.16/100 g), followed by bread containing 5% cricket meal (56.58/100 g) and bread fortified with 5% black soldier fly meal (48.82/100 g). The lowest protein content was observed in porridge fortified with 5% cricket meal (5.98/100 g). Carbohydrate content decreased with the increasing incorporation of edible insects (Table 1). The highest decrease in carbohydrate content was observed in the food product supplemented with Diptera, from 85.5/100 g (control) to 14.8/100 g (1–5% incorporation). While food products supplemented with Isoptera resulted in the lowest decrease in the carbohydrate content, 56.75/100 to 51.14/100 g (control and 1–5%, respectively). The food products supplemented with edible insects showed a significant increase in mineral composition when compared to the control (Table 2 and Appendix B). A study by Ogidi et al. (2025) presented the highest mineral composition, with the highest iron (818 mg/100 g) and zinc (378.04 mg/100 g) content reported in cookies fortified with cricket meal. The food products that retained the most nutrients, including protein, zinc, and iron, are bakery products, including bread, muffins, and biscuits.

### Consumer acceptance

Out of the 73 research papers reviewed, only 12 reported on consumer acceptance. Out of these, 58% reported high acceptance, and 42% moderate acceptance of the food product supplemented with edible insects. However, the acceptance of the food products supplemented with edible insects was mostly accepted with recommendations to mask the insects’ flavour (Table 3). Additionally, knowledge about the high nutritional value of food products supplemented with edible insects played a vital role in the acceptance of the food products. Bread, cookies, and energy bars were highly accepted, whereas porridge was moderately accepted. Most studies (90%) indicated that most consumers were concerned about the visibility of edible insects in some food products, whereas 15% of consumers were concerned about the colour change in the food products supplemented with edible insects, and, most importantly, ~85% advocated for the masking of the flavour of edible insects (Table 3).

## Discussion

The protein content of food products supplemented with edible insects increased with the increasing concentration of edible insects. Edible insects contain appreciable amounts of protein and, therefore, are a suitable choice for increasing the protein content of commonly consumed food products (Biró et al., 2020; Ogidi et al., 2025; Pauter et al., 2018). The increased protein content in these food products will ensure that people consume sufficient protein, meeting the daily protein requirements of Increasing protein content in people’s diets will mitigate the prevalence of protein deficiency-related disorders like stunted growth. Proteins are considered a basic nutritional requirement for the normal functioning of the human body (Johnson et al., 1999). Sufficient daily protein intake (0.8–1 g per kg of body weight a day) is essential for muscle protein synthesis, as it provides amino acids needed for muscle growth (Wolfe et al., 2008). Hence, the prevalence of stunted growth, wasting, and protein-energy malnutrition is due to the lack of protein in people’s diets, particularly in childhood, lactating women, and elderly individuals, will be mitigated (Endrinikapoulos et al., 2023). Monitoring measures are vital to track the effectiveness of the fortification, and before fortification, examination of the levels of nutrient inadequacy is important (Bourassa et al., 2023).

The carbohydrate content of different food products decreased with increasing concentrations of edible insects. According to Ayensu et al. (2019), the decrease in carbohydrate content in food products supplemented with a high concentration of edible insects could be attributed to the decrease in the amount of wheat flour used to produce the same final quantity and an increase in edible insect meal, which is lower in carbohydrates. This applies to maize, food products as well. Carbohydrates are one of the most important macronutrients, and they are broken down into glucose, making them the primary source of energy (Hlongwane et al., 2020; Holesh et al., 2023). However, in the human body, excess energy is stored as fat in the liver and muscle tissue (Ahmed et al., 2021; Norgan, 1997). Therefore, the carbohydrate concentration in the food should be carefully controlled to prevent having too much excess energy and fat stored (Holesh et al., 2023; Norgan, 1997; Sims & Danforth, 1987; Zhang et al., 2023).

Diets in disadvantaged communities mainly consist of starchy food products such as rice, bread, and maize meal porridges, which contribute to the prevalence of malnutrition (particularly protein and micronutrient deficiencies) in these communities (Ndunge Charles et al., 2024). Therefore, fortifying starchy food products with edible insects would reduce their carbohydrate content (including the available carbohydrates that tend to increase the glycaemic index of the food), while increasing the concentration of protein and micronutrients. Sugars in food products with a low glycaemic index are absorbed slowly, resulting in a person being full for longer, hence reducing the risk of obesity, diabetes, and other diabetes-related complications (Björck & Elmståhl, 2003; Sabarathinam, 2023; Sievenpiper, 2020). Thus, the decrease in carbohydrate concentration in foods fortified with edible insects is desirable because it reduces the risk of obesity and diabetes and other health conditions linked to high-carbohydrate diets.

Food products supplemented with edible insects had higher zinc and iron content compared to the control. The highest iron and zinc contents were reported in cookies supplemented with cricket meal. Therefore, in trying to mitigate the prevalence of mineral deficiencies, crickets could be the best edible insects to supplement staple food products for maximum mineral enhancement. Iron and zinc deficiency are common and problematic mineral deficiencies that are prevalent in females of reproductive age and children (Man et al., 2022; Nguyen et al., 2012; Prasad, 2020; Stein, 2010). Zinc and iron deficiencies give rise to retarded growth, slow wound healing, diarrhoea, and impaired skeletal structures (Prasad, 2020; Shahzad et al., 2014). Most staple foods consumed in developing countries, particularly in disadvantaged communities, are deficient in iron and zinc. Food products rich in zinc, such as meat, legumes, and dairy, are not easily accessible in rural communities because they are not readily available in these areas and are often sold at inflated prices in local tuckshops (Hambidge & Krebs, 2007). Therefore, consuming food products supplemented with edible insects can play an important role in reducing zinc and iron deficiencies worldwide.

The current review highlights that bakery food products were the most selected for the development of innovative food products fortified with edible insects. Bakery food products are well-accepted worldwide and are considered a staple food in developed countries (Wieczorek et al., 2022). They are a key part of daily diets in developed countries; hence, they were the most studied food products, as most studies were conducted in developed countries. Staple food products that are commonly consumed and accessible to most people in Africa include maize porridges, cassava, soups (e.g. egusi soup), and rice, which have rarely been used to develop food products fortified with edible insects (Indriani et al., 2020; Omotayo et al., 2020). However, most studies conducted in Nigeria, Kenya, and Ghana used bakery products instead of staple food products that are commonly consumed in African communities (Adeboye et al., 2016; Akande et al., 2020; Awobusuyi, Pillay, et al., 2020; Dewi et al., 2020; Duku et al., 2023; Ogidi et al., 2025; Ouma et al., 2022). Therefore, studies being conducted in developing countries should use staple foods that are commonly consumed and accessible to many people in these regions, particularly in disadvantaged communities where different forms of malnutrition are prevalent. Our review highlighted an increase in consumer acceptance of food products supplemented with edible insects in both developed and developing countries. This implies that people are more willing to consume insects when they are hidden as an ingredient in common food products. Previous studies have reported that incorporating edible insects into value-added food products will increase consumer acceptance and utilisation of edible insects (Acosta-Estrada et al., 2021; García-Segovia et al., 2020).

The current review showed that many of the studies were conducted in developed countries where the consumption of edible insects is not well accepted because of barriers and food neophobia, such as fear and disgust associated with eating insects (Olivadese & Dindo, 2023). However, the incorporation of edible insect meal into already popular food products contributed to the acceptance of the food products supplemented with edible insects. To further ensure that the food products fortified with edible insects are utilised and introduced to broader consumers, an emphasis on health benefits, affordability, and sustainability of these food products is vital. Promoting food products fortified with edible insects as high-protein, nutrient-dense, environmentally friendly food products can play an important role in broadening the consumer acceptance of these food products (Lumanlan et al., 2022). The increases in consumer acceptance of food products supplemented with edible insects in developed countries indicate that people are more willing to try food products supplemented with edible insects because this improves the sensory attributes of edible insects and masks the insect flavour (Adámek et al., 2018; Mazurek et al., 2022). Knowledge about the nutritional value of edible insects positively affected the attitude and willingness to consume food products supplemented with edible insects. According to Baker et al. (2022), people are becoming aware of the nutritional value and quality of the food they consume. Therefore, consumer acceptance of food products supplemented with edible insects is dependent on how they are presented and on knowledge about their nutritional value. Policymakers can support adaptation by developing clear regulations, encouraging insect farming, and integrating fortified products into school feeding programs. However, continued research is needed to assess consumer perceptions, long-term health impacts, and supply chain sustainability. Together, these strategies can ease the transition toward wider acceptance and utilisation of insect-fortified foods.

## Conclusion

The addition of insect meals to various food products resulted in a significant increase in nutrient content, particularly protein, zinc, and iron, indicating that fortifying staple food products with edible insects can play a vital role in mitigating the risk of malnutrition. Protein, zinc, and iron concentrations increased significantly with an increase in insect meal concentration; on the other hand, carbohydrate content decreased with an increase in insect meal concentration. Bakery products were the most used food products because they are well accepted worldwide and considered a staple food in developed countries, where most of the studies reviewed were conducted. However, more work needs to be done in Africa, as there is limited research on African communities’ staple foods. Food products supplemented with edible insects were well-accepted when insects were invisible in the food product, and less accepted when organoleptic properties, especially taste, were not detectable. Therefore, formulation of the foods supplemented with insects should be optimised and coupled with strategies that promote the utilisation of insects as a source of food, changing the negative perception of some of the target consumers about insects, and nutrition education are some suggested strategies. Knowledge about the good nutritional value of the insects also increased their consumer acceptability. Overall, the study findings indicate that edible insects have a good potential for use as an economically and environmentally sustainable strategy for addressing food and nutrition insecurity in developing countries.

## Supplementary Material

Supplementary Materials

## Figures and Tables

**Table 1:** Nutrient composition (g/100 g) and energy (kJ) of foods fortified with edible insects at different levels of fortification (%).

Level of fortification with insects (%)								
Order	Nutrients	0%	1–5%	6–10%	11–15%	16–20%	20+%	Number of publications
Lepidoptera	Protein	13.96 ± 0.40	18.82 ± 0.43	20.78 ± 1.18	23.99 ± 0.32	25.83 ± 0.57	28.2 ± 0.01	
	Fat	15.46 ± 0.35	16.47 ± 0.33	23.88 ± 0.47	24.51 ± 0.36	25.41 ± 0.16	26.1 ± 0.02	
	Carbohydrates	40.15 ± 0.47	37.30 ± 0.20	49.99 ± 1.17	46.77 ± 0.26	43.40 ± 0.58	34.1 ± 0.06	8
	Fibre	4.20 ± 0.03	5.59 ± 0.12	1.47 ± 0.04	1.59 ± 0.07	1.94 ± 0.14	3.8 ± 0.02	
	Ash	0.95 ± 0.07	1.11 ± 0.05	1.14 ± 0.03	0.77 ± 0.16	1.51 ± 0.5	3.8 ± 0.02	
	Energy	293.15 ± 1.86	319.02 ± 1.86	466.6 ± 0.30	473.50 ± 0.60	476.6 ± 1.10	484.12 ± 0.06	
Blattodea	Protein	9.24 ± 0.36	23.39 ± 0.62	21.15 ± 0.24	28.39 ± 0.30	19.55 ± 0.42	21.35 ± 0.26	
	Fat	13.96 ± 0.30	14.19 ± 0.50	19.93 ± 0.35	21.13 ± 0.41	17.68 ± 0.61	17.01 ± 0.62	5
	Carbohydrates	–	–	–	–	–	–	
	Fibre	8.3 ± 0.5	13.2 ± 0.5	10.3 ± 0.4	13.00 ± 0.5	–	–	
	Ash	1.7 ± 0.5	3.5 ± 0.6	4.00 ± 0.5	4.2 ± 0.4	–	–	
	Energy	303.30 ± 3.00	–	322.40 ± 0.14	–	338.10 ± 0.40	358.80 ± 0.71	
Orthoptera	Protein	15.77 ± 1.08	15.99 ± 0.33	21.77 ± 0.58	15.82 ± 0.02	18.91 ± 1.49	22.28 ± 0.28	35
	Fat	17.78 ± 0.68	18.48 ± 0.49	18.19 ± 0.53	28.77 ± 0.28	20.75 ± 0.44	15.46 ± 0.34	
	Carbohydrates	53.24 ± 1.02	35.09 ± 0.76	47.83 ± 0.83	44.97 ± 0.44	39.78 ± 0.90	41.72 ± 0.47	
	Fibre	3.66 ± 0.06	2.71 ± 0.19	7.58 ± 0.65	5.98 ± 0.86	8.76 ± 0.51	5.05 ± 0.19	
	Ash	2.91 ± 0.09	2.58 ± 0.07	3.65 ± 0.01	–	9.48 ± 0.06	1.91 ± 0.66	
	Energy	914.92 ± 4.02	501.61 ± 5.88	997.82 ± 6.95	1389.50 ± 3.43	1316.33 ± 3.02	329.25 ± 4.45	
Coleoptera	Protein	9.48 ± 0.27	18.53 ± 0.44	13.18 ± 0.22	14.63 ± 0.24	16.03 ± 0.33	19.16 ± 0.42	20
	Fat	17.9 ± 0.35	16.15 ± 0.33	12.40 ± 0.20	23.77 ± 0.37	17.46 ± 0.39	22.22 ± 0.45	
	Carbohydrates	57.43 ± 1.11	41.03 ± 1.06	40.46 ± 0.48	40.89 ± 1.13	45.94 ± 0.53	38.85 ± 0.54	
	Fibre	2.71 ± 0.18	7.56 ± 0.45	2.54 ± 0.17	7.74 ± 0.82	1.96 ± 0.05	5.34 ± 0.01	
	Ash	1.07 ± 0.11	2.57 ± 0.04	1.18 ± 0.02	0.98 ± 0.03	1.20 ± 0.03	2.00 ± 0.04	
	Energy	512.20 ± 3.79	1469 ± 11.20	659.95 ± 5.41	501.38 ± 5.23	412.59 ± 1.83	455.52 ± 4.12	
Isoptera	Protein	10.00 ± 0.01	14.26 ± 0.17	–	–	–	–	
	Fat	12.52 ± 0.31	12.62 ± 0.33	–	–	–	–	
	Carbohydrates	56.75 ± 1.63	51.14 ± 3.07	–	–	–	–	2
	Fibre	10.34 ± 0.06	12.46 ± 0.20	–	–	–	–	
	Ash	3.15 ± 0.04	3.85 ± 0.15	–	–	–	–	
Diptera	Protein	12.69 ± 0.60	45.09 ± 0.82	–	–	–	–	1
	Fat	1.19 ± 0.02	35.82 ± 0.66	–	–	–	–	
	Carbohydrates	85.57 ± 0.58	14.84 ± 0.35	–	–	–	–	
	Ash	0.64 ± 0.01	4.25 ± 0.00	–	–	–	–	

(%) Percentages are the incorporation rates of edible insects. (–) means that there are no reports for those incorporation rates. Number of publications: the publications used are listed in Appendix A.

**Table 2:** Comparison of mineral content of food enriched with edible insects.

Level of fortification with edible insects (%)								
Order	Minerals	0%	1–5%	6–10%	11–15%	16–20%	20+%	Number of publications
Lepidoptera	Iron	4.49 ± 0.19	4.6 ± 0.31	4.79 ± 0.04	4.91 ± 0.04	4.98 ± 0.27	–	2
	Zinc (mg)	2.31 ± 0.01	2.90 ± 0.06	3.34 ± 0.13	3.37 ± 0.40	3.40 ± 0.14	9.31 ± 0.02	
Blattodea	Iron	3.69 ± 0.78	17.47 ± 0.63	30.20 ± 0.39	34.48 ± 0.43	36.85 ± 0.67	43.33 ± 0.93	3
	Zinc (mg)	3.06 ± 0.31	6.91 ± 0.42	8.88 ± 0.38	11.24 ± 0.44	8.6 ± 0.51	12.85 ± 0.74	
Orthoptera	Iron	5.25 ± 0.16	0.72 ± 0.05	5.65 ± 0.07	37.2 ± 0.08	52.1 ± 0.45	–	7
	Zinc (mg)	7.96 ± 0.05	1.40 ± 0.02	7.99 ± 0.05	28.9 ± 0.02	37.9 ± 0.13	–	
Coleoptera	Iron	20.77 ± 0.17	135.30 ± 0.01	143.5 ± 0.01	–	–	–	2
	Zinc (mg)	52.15 ± 0.01	363.6 ± 0.02	372.4 ± 0.01	–	–	–	
Diptera	Iron	12.69 ± 0.06	45.09 ± 0.82	–	–	–	–	1
Order	Zinc (mg)	1.19 ± 0.02	35.82 ± 0.66	–	–	–	–	

(%) Percentages are the incorporation rates of edible insects. (–) means that there are no reports for those incorporation rates. Number of publications: the publications used are listed in Appendix B.

**Table 3:** Consumer acceptance of food products enriched with edible insects.

Insect used	Product	Consumer acceptance	Key comments	Reference
Cricket meal	Complementary porridge	Moderate acceptance.	Enhancing the taste was recommended, but it was accepted by the caregivers as a suitable complementary food	Abonge et al., 2022
Cricket meal	Energy and protein bars	High acceptance.	It was not visually appealing; however, nutritional benefits contributed to their acceptance. Appearance was initially a concern but improved with flavour variety and labelling as ‘energy-boosting’.	Adàmek et al., 2018
Cricket meal	Bread and cookies	High acceptance.	Consumers were concerned about the visibility of the edible insects in bread.	Bawa et al., 2020
Cricket meal	Biscuits	High acceptance.	Younger children showed more willingness to try the food product, and they were more accepting of a decreased visibility of the edible insects.	Homann et al., 2017
Cricket meal	Whole wheat bread	High acceptance.	Knowledge of nutritional value plays a crucial role in the acceptance of food products.	Mafu et al., 2022
Cricket meal	Pork pate	High acceptance.	Consumer education about the nutritional value of edible insects is recommended to improve acceptance.	Roncolini et al., 2020
Palm weevil larvae meal	Cookies	Moderate acceptance.	Scepticism was initially noted; however, that changed with knowing that it is a good protein source and increased acceptance when labelled as a protein source.	Adeboye et al., 2016
Silkworm pupae, locusts	Biscuits	Moderate acceptance.	Knowledge about nutritional value improved acceptance, particularly with young adults who were more open to trying the product.	Akande et al., 2020
Palm weevil larvae	Biscuits	High acceptance.	Masking the insect flavour played a vital role in enhancing acceptability.	Ayensu et al., 2019
Sorghum and cricket meal	Biscuits	High acceptance.	Changes in the colour profile raised concerns and scepticism about trying the biscuits	Duku et al., 2023
Mealworm meal	Wheat bread	Moderate acceptance.	Masking the insects’ flavour was recommended, and improved acceptability with nutrient information	Gantner et al., 2022
Mealworm, buffalo worm, and cricket meal	Pancakes	Moderate acceptance.	Masking the flavour of edible insects improved acceptance in taste tests with additional sweeteners.	Mazurek et al., 2022

## Data Availability

Data will be made available upon reasonable request.
