# Supplementary material for: Nutritional value and consumer acceptance of food products fortified with edible insects: a systematic review
Source: Cogent Food Agric. Author manuscript; Available in PMC 2026 Apr 4. (PMC7618968; doi:10.1080/23311932.2025.2602907)
Supplement: Supplementary Materials [file EMS212931-supplement-Supplementary_Materials.pdf]

Appendix A: Nutrient composition (g/100 g) of foods fortified with edible insects at different incorporation rates (%)

Edible insects	Food product	Nutrients	0%	2%	5%	10%	Reference
Cricket	Muffin	Protein	11.84 ± 0.24	12.27 ± 0.18	14.58 ± 0.54	16.51 ± 0.23	Pauter et al., 2018
		Fat	23.46 ± 0.74	23.64 ± 0.08	24.39 ± 0.17	25.34 ± 0.75	
		Carbohydrate	44.29 ± 0.18	41.82 ± 0.49	38.17 ± 0.09	34.45 ± 0.37	
		Ash	1.00 ± 0.14	1.10 ± 0.14	1.33 ± 0.32	1.65 ± 0.21	
		Energy (kJ)	435.66 ± 8.28	429.12 ± 1.91	430.51 ± 0.27	431.90 ± 4.35	

Edible insect	Food product	Nutrients	0%	2%	6%	10%	Reference
Cricket	Muffin	Protein	7.8 ± 0.55	7.63 ± 0.53	9.07 ± 0.64	10.45 ± 0.73	Zielinska et al., 2021
		Fat	15.32 ± 0.19	13.55 ± 0.52	15.17 ± 0.17	16.42 ± 0.48	
		Carbohydrate	51.17 ± 0.85	45.96 ± 0.72	47.74 ± 0.23	45.47 ± 0.58	
		Ash	0.9 ± 0.05	0.82 ± 0.08	0.93 ± 0.06	1.0 ± 0.09	
		Energy (kJ)	1568 ± 8.0	1412 ± 12.22	1527 ± 15.56	1558 ± 13.74	

Edible insect	Food product	Nutrients	0%	10%	20%	30%	Reference
Cricket	Pancakes	Protein	8.13 ± 0.19	10.48 ± 0.44	13.05 ± 0.34	15.66 ± 0.66	Mazurek et al., 2022
		Fat	7.93 ± 0.1	8.50 ± 0.19	9.54 ± 0.29	10.46 ± 0.18	
		Carbohydrate	34.23 ± 0.88	29.64 ± 0.55	24.69 ± 0.88	19.94 ± 0.26	
		Fibre	1.13 ± 0.06	1.32 ± 0.01	1.51 ± 0.01	1.70 ± 0.08	
		Ash	0.98 ± 0.05	0.99 ± 0.56	1.12 ± 0.04	1.21 ± 0.06	
		Energy (kJ)	255.32 ± 3.1	260.87 ± 0.9	260.87 ± 2.7	260.87 ± 0.66	

Edible insects	Food product	Nutrients	0%	2.5%	5%	7.5%	Reference
Cricket	Meat	Protein	14.46 ± 0.04	14.81 ± 0.08	16.87 ± 0.13	17.87 ± 0.13	Cavalheiro et al., 2023
		Fat	20.73 ± 0.48	20.32 ± 0.41	20.55 ± 0.23	21.69 ± 0.08	
		Ash	2.38 ± 0.01	2.28 ± 0.03	2.41 ± 0.02	2.50 ± 0.02	

Edible insect	Food product	Nutrients	0%	8%	16%	24%	Reference
Cricket	Snacks	Protein	0.45 ± 0.05	5.55 ± 0.07	10.70 ± 0.14	15.85 ± 0.21	Akullo et al., 2016

Edible insect	Food product	Nutrients	0%	2%	6%	10%	Reference
Cricket	Snacks	Ash	1.80 ± 0.03	1.95 ± 0.04	2.02 ± 0.06	2.17 ± 0.04	Smarzynski et al., 2019

Edible insect	Food product	Nutrients	0%				Reference
Cricket	Biscuits	Protein	9.48	11.22	12.97	14.71	Biró et al., 2020
		Fat	23.69	24.02	24.35	24.68	
		Carbohydrate	38.27	36.67	35.06	33.46	
		Fibre	7.00	6.66	6.32	5.98	
		Energy (kJ)	410.68	413.50	416.32	419.14	

Edible insects	Food product	Nutrients	0%	10%	30%	Reference
Cricket	Bread	Protein	14.69 ± 0.19	26.89 ± 0.76	27.72 ± 0.59	Osimani et al., 2018
		Fat	0.10 ± 0.01	1.53 ± 0.04	6.38 ± 0.28	
		Fibre	0.45 ± 0.01	2.44 ± 0.11	2.58 ± 0.05	
		Ash	0.69 ± 0.01	1.52 ± 0.01	1.57 ± 0.01	
		Energy (kJ)	259.50 ± 0.97	281.57 ± 8.08	262.99 ± 11.01	

Edible insect	Food Product	Nutrients	0%	5%	Reference
Cricket	Porridge	Protein	4.28	5.98	Kinyuru et al., 2021
		Fat	8.75	9.21	
		Carbohydrate	43.18	40.75	
		Fibre	3.24	3.34	
		Energy (kJ)	251.07	251.41	

Edible insect	Food Product	Nutrients	0%	5%	Reference
Cricket	Bread	Protein	12.69 ± 0.60	56.58 ± 0.86	González et al., 2019
		Fat	1.19 ± 0.02	27.08 ± 0.72	
		Carbohydrate	85.57 ± 0.58	12.33 ± 0.41	
		Ash	0.64 ± 0.01	4.02 ± 0.01	

Edible insects	Food product	Nutrients	0%	10%	Reference
Cricket	Cookies	Protein	8.47 ± 0.01	11.70 ± 0.00	Ogidi et al., 2025
		Fat	14.04 ± 0.61	13.20 ± 0.65	
		Carbohydrate	57.16 ± 1.20	53.80 ± 2.10	
		Fibre	9.63 ± 0.10	11.31 ± 0.40	
		Ash	2.30 ± 0.00	3.60 ± 0.30	

(*Continued*)

Continued.

Edible insect	Food product	Nutrients	0%	10%	Reference
Cricket	Bread	Protein	16.91 ± 0.55	21.13 ± 0.25	Bawa et al., 2020
		Fat	11.99 ± 1.11	14.49 ± 0.28	
		Carbohydrate	68.86 ± 0.54	61.63 ± 0.48	
		Fibre	0.14 ± 0.03	0.77 ± 0.07	
		Ash	2.25 ± 0.02	2.76 ± 0.01	

Edible insect	Food product	Nutrients	0%	10%	Reference
Cricket	Cookies	Protein	9.45 ± 0.73	15.79 ± 0.09	Bawa et al., 2020
		Fat	24.66 ± 2.97	23.31 ± 0.79	
		Carbohydrate	64.49 ± 1.84	59.79 ± 0.84	
		Fibre	0.10 ± 0.00	0.64 ± 0.07	
		Ash	1.41 ± 0.41	1.09 ± 0.06	

Edible insect	Food product	Nutrients	0%	10%	Reference
Cricket	Biscuits	Protein	9	12	Homann et al., 2017
		Fat	37	38	
		Carbohydrate	51	53	
		Fibre	1.7	2.4	
		Ash	1.9	2.2	
		Energy (kJ)	1.94	1.9	

Edible insects	Food product	Nutrients	0%	5%	10%	15%	20%	25%	Reference
Cricket	Biscuits	Protein	09.64 ± 0.21	11.61 ± 0.56	13.43 ± 0.38	16.45 ± 0.34	19.90 ± 0.44	21.66 ± 0.40	Niaba Koffi et al., 2013
		Fat	21.51 ± 0.43	20.26 ± 0.87	0.08 ± 0.66	19.99 ± 0.75	19.59 ± 0.84	19.03 ± 0.92	

Edible insect	Food product	Nutrients	0%	10%	15%	20%	25%	30%	Reference
Cricket	Bread	Protein	15.75 ± 3.57	18.97 ± 0.37	21.90 ± 0.46	22.79 ± 0.07	25.86 ± 0.95	28.46 ± 0.01	Mafu et al.,2022
		Carbohydrate	73.53 ± 0.07	68.03 ± 0.76	65.55 ± 0.72	63.01 ± 1.79	58.04 ± 0.34	56.65 ± 1.39	

Edible insect	Food product	Nutrients	0%	2%	6%	10%	Reference
Cricket	Snacks	Fat	3.4 ± 0.4	3.3 ± 0.9	2.6 ± 0.5	2.2 ± 0.5	Kowalski et al., 2022
		Ash	1.49 ± 0.01	1.56 ± 0.01	1.73 ± 0.01		

Edible insect	Food product	Nutrients	0%	2.5%	5%	7.5%	Reference
Cricket	Meat	Protein	14.46 ± 0.0	14.81 ± 0.08	16.87 ± 0.13	17.87 ± 0.13	Cavalheiro et al., 2023
		Fat	20.73 ± 0.4	20.32 ± 0.41	20.55 ± 0.23	21.69 ± 0.08	
		Ash	2.38 ± 0.01	2.28 ± 0.03	2.41 ± 0.02	2.50 ± 0.02	

Edible insect	Food product	Nutrients	0%	10%	15%	20%	Reference
Cricket	Biscuits	Protein	10.90 ± 0.04	14.3 ± 0.01	15.2 ± 0.01	17.4 ± 0.02	Duku et al., 2023
		Fat	17.3 ± 0.04	17.8 ± 0.03	21.00 ± 0.02	22.7 ± 0.02	
		Carbohydrate	66.6 ± 0.04	61.9 ± 0.04	59.0 ± 0.01	52.3 ± 0.03	
		Ash	1.1 ± 0.01	1.45 ± 0.03	1.47 ± 0.05	2.00 ± 0.02	
		Energy (kJ)	3192 ± 0.57	3262 ± 0.47	3282 ± 0.34	3335 ± 0.29	

Edible insect	Food product	Nutrients	0%	5%	7.5%	10%	Reference
Cricket	Cookies	Protein	3.9 ± 0.45	8.9 ± 0.27	11.9 ± 0.32	13.3 ± 0.29	Aleman et al., 2022
		Fat	33.8 ± 1.34	31.5 ± 1.05	30.2 ± 1.34	28.5 ± 1.07	
		Carbohydrate	48.1 ± 1.33	44.4 ± 1.89	41.3 ± 1.33	39.8 ± 1.89	
		Ash	2.9 ± 0.03	2.8 ± 0.02	2.6 ± 0.03	2.5 ± 0.02	

Edible insect	Food product	Nutrients	0%	3%	6%	9%	12%	15%	References
Cricket	Cookies	Protein	15.00 ± 0.08	26.28 ± 0.09	28.98 ± 0.06	31.10 ± 0.04	32.78 ± 0.04	36.31 ± 0.06	Baik et al., 2025
		Fat	15.84 ± 0.50	17.16 ± 0.61	20.83 ± 0.56	22.35 ± 0.51	23.28 ± 0.10	24.20 ± 0.6	
		Ash	1.60 ± 0.01	1.69 ± 0.01	1.77 ± 0.00	1.84 ± 0.00	1.95 ± 0.02	2.05 ± 0.01	

Edible insect	Food product	Nutrients	0%	5%	10%	15%	References
Cricket	Biscuits	Protein	9.5±0.08	11.8±0.21	13.1±0.08	16.1±0.21	Arama, 2025
		Fat	18.6±0.40	20.6±0.10	20.9±0.31	22.4±0.40	
		Carbohydrates	68.9±2.45	63.9±3.12	61.4±2.45	56.5±3.12	
		Fibre	0.6±0.02	1.1±0.02	1.5±0.02	1.8±0.02	
		Ash	0.3±0.03	1.0±0.07	1.1±0.31	1.1±0.09	

(*Continued*)

Continued.

Edible insect	Food product	Nutrients	0%	5%	10%	15%	References
Cricket	Rice noodles	Protein	4.96 ± 0.15	13.31 ± 0.59	22.16 ± 1.10	24.61 ± 0.13	H. Li et al., 2025
		Fat	2.05 ± 0.01	1.92 ± 0.01	4.06 ± 0.07	6.36 ± 0.03	
		Carbohydrates	89.80 ± 0.17	82.70 ± 0.57	70.13 ± 1.16	64.42 ± 0.15	
		Fibre	1.42 ± 0.01	1.46 ± 0.03	2.38 ± 0.02	3.15 ± 0.05	
		Ash	1.76 ± 0.01	0.60 ± 0.02	1.27 ± 0.01	1.46 ± 0.03	

Edible insect	Food product	Nutrients	0%	5%	10%	15%	References
Cricket	Crackers	Protein	14.83 ± 0.05	15.99 ± 0.03			Ivanišová et al., 2023
		Fat	30.82 ± 0.97	33.93 ± 0.02			
		Ash	1.37 ± 0.05	1.48 ± 0.02			
		Energy	570.7 ± 0.58	573.9 ± 1.19			

Edible insect	Food product	Nutrients	0%	5%	10%	15%	20%	25%	30%	35%	40%	50%	60%	70%	80%	90%	Reference
Cricket	Bread	Protein	11.6	14.52	17.44	20.36	23.28	26.2	29.12	32.04	34.96	40.8	46.64	52.48	58.32	64.16	Orkusz & Orkusz, 2025
		Fat	1.8	2.64	3.48	4.31	5.15	5.99	6.83	7.66	8.5	10.18	11.85	13.53	15.2	16.88	
		Carbohydrates	68	64.63	61.25	57.88	54.5	51.13	47.75	44.38	41	34.25	27.5	20.75	14	7.25	
		Fibre	2.9	3.23	3.56	3.89	4.22	4.55	4.88	5.21	5.54	6.2	6.86	7.52	8.18	8.84	
		Energy (kJ)	342	347.68	353.35	359.03	364.7	370.38	376.05	381.73	387.4	398.75	410.1	421.45	432.8	444.15	

Edible insects	Food product	Nutrients	0%	2%	6%	10%	Reference
Mealworm	Muffins	Protein	7.8 ± 0.55	7.9 ± 0.55	9.16 ± 0.64	8.63 ± 0.6	Zielinska et al., 2021
		Fat	15.32 ± 0.19	14.14 ± 0.38	16.68 ± 0.13	16.39 ± 0.1	
		Carbohydrate	51.17 ± 0.85	47.75 ± 0.67	47.43 ± 0.55	44.0 ± 0.42	
		Ash	0.9 ± 0.05	0.88 ± 0.07	0.95 ± 0.05	0.98 ± 0.05	
		Energy (kJ)	1569 ± 8.0	1469 ± 11.2	1527 ± 15.56	1558 ± 13.74	

Edible insect	Food product	Nutrients	0%	10%	20%	30%	Reference
Mealworm	Pancake	Protein	8.13 ± 0.19	9.29 ± 0.54	10.53 ± 0.68	11.73 ± 0.38	Mazurek et al., 2022
		Fat	7.93 ± 0.1	8.70 ± 0.37	9.96 ± 0.45	11.10 ± 0.1	
		Carbohydrate	34.23 ± 0.88	29.73 ± 0.43	25.05 ± 0.33	20.55 ± 0.77	
		Fibre	1.13 ± 0.06	1.22 ± 0.05	1.27 ± 0.05	1.33 ± 0.04	
		Ash	0.98 ± 0.05	0.91 ± 0.02	0.87 ± 0.04	0.86 ± 0.04	
		Energy (kJ)	255.32 ± 3.1	263.74 ± 2.4	269.66 ± 2.3	275.86 ± 2.6	

Edible insect	Food product	Nutrients	0%	10%	20%	30%	Reference
Mealworm beetle	Cookies	Protein	9.4 ± 0.01	11.08 ± 0.06	12.80 ± 0.03	14.73 ± 0.02	Sriprablom et al., 2022
		Fat	21.98 ± 0.29	23.57 ± 0.20	25.45 ± 0.36	26.33 ± 0.44	
		Carbohydrate	64.94 ± 0.28	60.54 ± 0.40	56.16 ± 0.51	52.91 ± 0.49	
		Ash	1.49 ± 0.01	1.59 ± 0.02	1.74 ± 0.01	1.84 ± 0.01	
		Energy (kJ)	495.21 ± 1.4	498.60 ± 0.43	504.88 ± 1.25	507.45 ± 1.99	

Edible insect	Food product	Nutrients	0%	13%	17%	20%	Reference
Mealworm	Biscuits	Protein	7.8 ± 0.2	16.11 ± 0.09	19.00 ± 0.07	21.82 ± 0.51	Ortolá et al., 2022
		Fat	20.8 ± 0.3	13.06 ± 0.06	15.32 ± 0.07	16.59 ± 0.12	

Edible insects	Food product	Nutrients	0%	15%	20%	30%	Reference
Mealworm	Biscuits	Protein	9.09 ± 0.46	10.82 ± 0.5	11.97 ± 0.5	13.52 ± 0.6	Zielinska & Pankiewicz, 2020
		Fat	27.03 ± 1.48	28.47 ± 0.36	26.97 ± 1.69	27.17 ± 0.39	
		Carbohydrate	63.6 ± 1.63	60.27 ± 1.72	60.36 ± 1.49	58.69 ± 1.63	

Edible insect	Food product	Nutrients	0%	15%	30%	Reference
Mealworm	Snacks	Protein	10.78 ± 0.02	13.48 ± 0.04	15.51 ± 0.04	Kowalski et al., 2022
		Fat	36.20 ± 0.06	32.91 ± 0.08	26.69 ± 0.11	

Edible insect	Food product	Nutrients	0%	10%	20%	Reference
Mealworm	Cake	Protein	5.08 ± 0.03	6.32 ± 0.03	8.04 ± 0.03	Kowalski et al., 2022
		Fat	0.58 ± 0.03	1.93 ± 0.01	3.50 ± 0.06	
		Ash	0.22 ± 0.01	0.3 ± 0.01	0.46 ± 0.00	

Edible insects	Food products	Nutrients	0%	10%	30%	References
Mealworm	Snacks	Protein	12.53 ± 1.06	15.59 ± 0.13	24.98 ± 3.27	Roncolini et al., 2020
		Fat	0.13 ± 0.01	0.48 ± 0.10	5.34 ± 0.01	
		Fibre	3.27 ± 0.10	4.38 ± 0.50	6.36 ± 1.19	
		Ash	0.55 ± 0.04	0.77 ± 0.02	1.37 ± 0.07	
		Energy (kJ)	327.61 ± 12.44	330.26 ± 3.75	351.51 ± 10.43	

(*Continued*)

Continued.

Edible insect	Food product	Nutrients	0%	15%	30%	Reference
Mealworm	Bread	Protein	10.78 ± 0.02	13.23 ± 0.15	14.06 ± 0.01	Kowalski et al., 2022
		Fat	36.20 ± 0.06	34.07 ± 0.01	32.62 ± 0.16	
		Ash	1.49 ± 0.01	1.52 ± 0.01	1.57 ± 0.00	

Edible insect	Food product	Nutrients	0%	5%	Reference
Mealworm	Bread	Protein	12.69 ± 0.60	45.09 ± 0.82	González et al., 2019
		Fat	1.19 ± 0.02	35.82 ± 0.66	
		Carbohydrate	85.57 ± 0.58	14.84 ± 0.35	
		Ash	0.64 ± 0.01	4.25 ± 0.00	

Edible insect	Food products	Nutrients	0%	5%	10%	15%	Reference
Mealworm	Bread	Protein	11.30 ± 0.12	14.13 ± 0.11	16.95 ± 0.16	19.77 ± 0.04	Gantner et al., 2022
		Fat	2.23 ± 0.04	3.07 ± 0.01	3.92 ± 0.02	4.77 ± 0.06	
		Carbohydrate	62.09 ± 0.45	59.10 ± 1.89	56.10 ± 0.49	53.11 ± 1.05	
		Fibre	7.47 ± 0.87	7.56 ± 0.45	7.65 ± 0.61	7.74 ± 0.82	

Edible insect	Food product	Nutrients	0%	5%	10%	15%	Reference
Mealworm	Bread	Protein	9.63 ± 0.01	12.63 ± 0.16	13.21 ± 0.21	13.73 ± 0.04	Khuenpet et al., 2020
		Ash	1.49 ± 0.01	1.56 ± 0.01	1.73 ± 0.01		

Edible insect	Food product	Nutrients	0%	15%	Reference
Mealworm	Muffin	Protein	5.85 ± 0.82	11.70 ± 0.81	Çabuk, 2021
		Fat	31.21 ± 1.66	36.56 ± 1.26	
		Carbohydrate	34.56 ± 4.02	22.07 ± 1.63	

Edible insect	Food product	Nutrients	0%	5%	References
Mealworm	Crackers	Protein	14.83 ± 0.05	15.42 ± 0.04	Ivanišová et al., 2023
		Fat	30.82 ± 0.97	32.88 ± 0.11	
		Ash	1.37 ± 0.05	1.56 ± 0.03	
		Energy	570.7 ± 0.58	579.1 ± 3.33	

Edible insect	Food product	Nutrients	0%	5%	10%	15%	References
Termites	Biscuits	Protein	10.5 ± 0.4	36.4 ± 0.4	38.3 ± 0.5	41.0 ± 0.40	Awobusuyi et al., 2020
		Fat	14.3 ± 0.4	22.3 ± 0.5	25.2 ± 0.4	28.2 ± 0.4	
		Fibre	8.3 ± 0.5	13.2 ± 0.5	10.3 ± 0.4	13 ± 0.5	
		Ash	1.7 ± 0.5	3.5 ± 0.6	4.0 ± 0.5	4.2 ± 0.4	

Edible insect	Food product	Nutrients	0%	8%	16%	24%	Reference
Termites	Snacks	Protein	0.45 ± 0.05	5.55 ± 0.07	10.70 ± 0.14	15.85 ± 0.21	Akullo et al., 2016

Edible insect	Food product	Nutrients	0%	5%	Reference
Termites	Bread	Protein	10.60 ± 0.90	15.63 ± 1.24	Kinyuru et al., 2009

Edible insect	Food product	Nutrients	0%	3%	5%	7%	10%	Reference
Grasshopper	Sausages	Protein	10.35 ± 0.1	15.37 ± 0.27	13.34 ± 0.10	12.79 ± 0.10	12.25 ± 0.17	Crus-Lopez et al., 2022
		Fat	13.94 ± 0.3	14.27 ± 0.13	13.36 ± 0.25	13.68 ± 0.02	13.01 ± 0.07	
		Ash	2.53 ± 0.10	3.41 ± 0.02	3.05 ± 0.02	3.19 ± 0.04	3.21 ± 0.01	

Edible insects	Food product	Nutrients	0%	5%	7%	10%	Reference
Grasshopper	Biscuits	Protein	10.61 ± 0.18	14.28 ± 0.28	15.36 ± 0.51	16.45 ± 0.84	Dewi et al., 2020
		Fat	19.98 ± 0.45	19.68 ± 1.57	21.97 ± 1.56	22.25 ± 0.49	
		Carbohydrate	60.09 ± 0.54	56.95 ± 1.33	54.71 ± 1.20	52.12 ± 0.73	
		Fibre	6.23	11.03	15.55	19.75	
		Ash	7.31	5.69	4.30	5.80	
		Energy (kJ)	462.63 ± 4.99	462.10 ± 9.12	478.01 ± 8.01	474 ± 5.14	

Edible insect	Food product	Nutrients	0%	15%	30%	Reference
Grasshopper	Snacks	Protein	10.78 ± 0.02	13.11 ± 0.12	13.69 ± 0.16	Jimenez et al., 2020
		Fat	36.20 ± 0.06	35.48 ± 0.23	29.35 ± 0.23	

Edible insect	Food product	Nutrients	0%	15%	30%	Reference
Grasshopper	Bread	Protein	11.2 ± 11	14.8 ± 4	17.4 ± 6	Haber et al., 2019
		Fat	10 ± 1	18 ± 1	30 ± 1	
		Fibre	12 ± 3	13 ± 3	16 ± 1	
		Ash	19 ± 0.1	24 ± 0.1	26 ± 0.1	

(*Continued*)

Continued.

Edible insect	Food product	Nutrients	0%	15%	Reference
Grasshopper	Muffin	Protein	5.85 ± 0.82	12.91 ±	Çabuk, 2021
		Fat	31.21 ± 1.66	36.47 ± 0.75	
		Carbohydrate	34.56 ± 4.02	21.87 ± 0.58	
		Energy (kJ)	442.54 ± 2.62	467.37 ± 6.52	

Edible insect	Food product	Nutrients	0%	5%	10%	References
Grasshopper	Rice	Protein	7.32	9.93	9.18	Olawale et al., 2024
		Fat	38.89	18.85	27.77	
		Carbohydrates	35.99	55.07	48.02	
		Fibre	2.55	4.83	3.76	
		Ash	3.68	4.23	5.52	
		Energy	2189	1798	2003	

Edible insect	Food product	Nutrients	0%	5%	References
Grasshopper	Crackers	Protein	14.83 ± 0.05	15.23 ± 0.07	Ivanišová et al., 2023
		Fat	30.82 ± 0.97	34.97 ± 0.62	
		Ash	1.37 ± 0.05	1.41 ± 0.02	
		Energy	570.7 ± 0.58	585.4 ± 3.12	

Edible insects	Food product	Nutrient	0%	5%	10%	15%	20%	Reference
Imbrasia oyemensis	Cookies	Protein	10.1 ± 0.3	12.3 ± 0.7	13.32 ± 0.8	16.4 ± 0.2	17.5 ± 0.6	Aboubacar et al., 2022
		Fat	15.1 ± 0.4	16.4 ± 0.6	17.07 ± 0.3	17.5 ± 0.2	18.2 ± 0.1	
		Carbohydrate	68.93 ± 0.04	66.44 ± 0.1	64.58 ± 0.12	62.7 ± 0.03	60.7 ± 0.2	
		Fibre	0.1 ± 0.06	1.05 ± 0.02	1.1 ± 0.01	1.14 ± 0.08	1.2 ± 0.05	
		Ash	0.57 ± 0.10	0.76 ± 0.03	1.1 ± 0.05	1.4 ± 0.05	1.66 ± 0.8	
		Energy (kJ)	453.4 ± 1.20	464.3 ± 0.7	466.6 ± 0.3	473.5 ± 0.6	476.6 ± 1.1	

Edible insect	Food product	Nutrients	0%	5%	10%	15%	20%	Reference
Gonimbrasia zambesina	Muffin	Protein	19.67 ± 0.70	25.47 ± 0.50	28.23 ± 1.55	31.58 ± 0.44	34.15 ± 0.54	Ouma et al., 2022
		Fat	28.83 ± 0.64	29.75 ± 0.38	30.68 ± 0.64	31.52 ± 0.51	32.62 ± 0.22	
		Carbohydrate	47.00 ± 1.16	39.96 ± 0.37	35.40 ± 2.22	30.83 ± 0.49	26.09 ± 0.96	
		Fibre	0.89 ± 0.00	1.14 ± 0.00	1.82 ± 0.07	2.04 ± 0.06	2.67 ± 0.22	
		Ash	0.93 ± 0.09	1.11 ± 0.11	1.18 ± 0.01	0.23 ± 0.26	1.35 ± 0.20	

Edible insect	Food product	Nutrients	0%	10%	20%	30%	Reference
Darkling beetle	Cookies	Protein	9.4 ± 0.01	11.07 ± 0.08	13.01 ± 0.06	14.92 ± 0.05	Sripablom et al., 2022
		Fat	21.98 ± 0.29	22.09 ± 0.75	24.68 ± 0.09	25.80 ± 0.45	
		Carbohydrate	64.94 ± 0.28	63.99 ± 0.53	57.73 ± 0.22	53.84 ± 0.44	
		Ash	1.49 ± 0.01	1.68 ± 0.02	1.74 ± 0.04	1.83 ± 0.02	
		Energy (kJ)	495.21 ± 1.4	499.05 ± 0.52	505.08 ± 0.33	505.92 ± 2.53	

Edible insect	Food product	Nutrients	0%	10%	20%	30%	Reference
Bombay locust	Cake	Protein	14.94 ± 0.58	18.40 ± 0.59	20.83 ± 0.15	24.94 ± 0.30	Indriani et al., 2020
		Fat	21.55 ± 0.57	22.17 ± 0.36	21.49 ± 0.41	21.90 ± 0.64	
		Carbohydrate	21.08 ± 1.84	19.42 ± 1.36	19.10 ± 0.89	14.26 ± 0.48	
		Ash	1.26 ± 0.08	1.29 ± 0.06	1.33 ± 0.04	1.35 ± 0.02	
		Energy (kJ)	339.94 ± 3.62	350.83 ± 6.32	353.13 ± 6.06	354.33 ± 5.86	

Edible insect	Food Product	Nutrients	0%	2%	6%	10%	Reference
Buffalo worm	Snacks	Fat	3.4 ± 0.4	3.3 ± 0.9	2.6 ± 0.5	2.2 ± 0.5	Kowalski et al., 2022
		Ash	1.49 ± 0.01	1.56 ± 0.01	1.73 ± 0.01		

Edible insect	Food product	Nutrients	0%	5%	10%	15%	20%	References
Mopane worm	Muffin	Protein	19.67 ± 0.70	25.47 ± 0.50	28.23 ± 1.55	31.58 ± 0.44	34.15 ± 0.54	Moth, 2023
		Fat	28.83 ± 0.64	29.75 ± 0.38	30.68 ± 0.64	31.52 ± 0.51	32.62 ± 0.22	
		Carbohydrates	0.93 ± 0.09	1.11 ± 0.11	1.18 ± 0.01	1.23 ± 0.26	1.35 ± 0.20	
		Fibre	0.89 ± 0.00	1.14 ± 0.0	1.82 ± 0.07	2.04 ± 0.06	2.67 ± 0.22	
		Ash	47.00 ± 1.16	39.96 ± 0.37	35.40 ± 2.22	30.83 ± 0.49	26.09 ± 0.96	
		Energy	97.32 ± 0.18	97.44 ± 0.14	97.31 ± 0.08	97.19 ± 0.19	96.87 ± 0.19	

Edible insect	Food product	Nutrients	0%	5%	Reference
Mopane worm	Porridge (sorghum)	Protein	4.4	9.4	Ledbetter et al., 2024
Mopane worm	Porridge (millet)	Protein	4.3	9.3	

(*Continued*)

Continued.

Edible insect	Food Product	Nutrients	0%	35%	70%	Reference
Palm weevil	Biscuits	Protein	8.01 ± 0.05	9.63 ± 0.08	11.68 ± 0.42	Ayensu et al., 2019
		Fat	23 ± 0.5	34 ± 2.44	33 ± 2.08	
		Carbohydrate	57.49 ± 1.29	43.07 ± 2.6	32.88 ± 1.86	
		Fibre	3 ± 0.4	4 ± 0.00	5 ± 1.0	
		Ash	1.61 ± 0.01	1.64 ± 0.01	2.10 ± 0.01	
		Energy (kJ)	468.97 ± 0.65	516 ± 7.38	577.12 ± 15.15	
Edible insect	Food Product	Nutrients	0%	5%		Reference
Palm weevil	Cookies	Protein	8.47 ± 0.01	12.00 ± 0.80		Ogidi et al., 2025
		Fat	14.04 ± 0.61	13.10 ± 0.00		
		Carbohydrate	57.16 ± 1.20	53.60 ± 0.85		
		Fibre	9.63 ± 0.10	11.31 ± 0.04		
		Ash	2.30 ± 0.00	3.50 ± 0.11		
Edible insect	Food product	Nutrients	0%	5%		Reference
Black soldier fly	Bread	Protein	12.69 ± 0.60	48.82 ± 0.76		González et al., 2019
		Fat	1.19 ± 0.02	30.69 ± 0.80		
		Carbohydrate	85.57 ± 0.58	16.24 ± 0.63		
		Ash	0.64 ± 0.01	4.25 ± 0.01		

Edible insect	Food product	Nutrients	0%	5%	10%	15%	References
Silkworm	Rice noodles	Protein		12.06 ± 0.28	17.81 ± 0.08	25.85 ± 1.84	H. Li et al., 2025
		Fat		4.28 ± 0.01	4.47 ± 0.04	10.26 ± 0.07	
		Carbohydrates		80.62 ± 0.24	74.22 ± 0.07	55.50 ± 1.91	
		Fibre		1.57 ± 0.01	2.56 ± 0.03	3.74 ± 0.03	
		Ash		1.85 ± 0.03	2.45 ± 0.01	4.65 ± 0.01	
Edible insect	Food product	Nutrients	0%	5%	15%	25%	Reference
Caterpillar	Rice	Protein	7.32	9.09	9.06	12.09	Olawale et al., 2024
		Fat	38.89	28.86	23.72	28.11	
		Carbohydrates	35.99	50.83	53.36	44.77	
		Fibre	2.55	0.25	3.51	3.68	
		Ash	3.68	3.09	3.58	3.82	
		Energy	2189	2089.6	1937.9	2010	

Note: Values are mean ± Standard deviation of food products fortified with edible insects, % (percentages) on the top are incorporation rates of insect meal.

Appendix B: Comparison of mineral content (mg/100 g) of food enriched with edible insects at different incorporation rates (%)

Edible insects	Food product	Minerals	0%	10%	Reference
Cricket	Cookies	Iron	0.87 ± 0.32	1.37 ± 0.13	Bawa et al., 2020
		Phosphorus	82.1 ± 1.72	143.14 ± 2.39	
	Food product	Minerals	0%	10%	Reference
Cricket	Bread	Iron	1.55 ± 0.02	3.28 ± 1.47	Bawa et al., 2020
		Phosphorus	156.72 ± 1.16	221.66 ± 2.15	
	Food product	Minerals			Reference
Cricket	Cookies	Iron	109.63 ± 7.00	426.19 ± 22.80	Ogidi et al., 2025
		Zinc	205.00 ± 9.04	345.80 ± 10.82	
		Potassium	217.44 ± 0.04	317.50 ± 18.03	
	Food product	Minerals			Reference
Cricket	Cookies	Iron	378.04 ± 11.45	818.00 ± 30.05	Ogidi et al., 2025
		Zinc	412.02 ± 8.03	601.04 ± 18.18	
		Potassium	448.76 ± 32.11	573.81 ± 43.80	
	Food product	Minerals			Reference
Cricket	Porridge	Iron	9.86 ± 2.08	8.56 ± 1.45	Maiyo et al., 2022
		Zinc	1.86 ± 0.04	3.23 ± 0.28	
		Phosphorus	221.63 ± 5.57	372.71 ± 19.14	

(*Continued*)

Continued.

Edible insect	Food product	Minerals	0%	5%	Reference
Cricket	Bread	Iron	5.04	5.64	Kinyuru et al., 2021
	Food product	Minerals			Reference
Cricket	Cookies	Iron	109.63 ± 7.00	507.71 ± 8.04	Ogidi et al., 2025
		Zinc	205.00 ± 9.04	335.00 ± 9.0	
		Potassium	217.44 ± 0.04	311.32 ± 8.91	
	Food product	Minerals	0%	10%	Reference
Cricket	Biscuit	Iron	1.0	1.6	Homann et al., 2017
		Zinc	1.1	3.1	

	Food product	Minerals	0%	10%	15%	20%			Reference
Cricket	Biscuits	Iron	23.3	30.5	37.2	52.1			Duku et al., 2023
		Zinc	29.45	34	28.9	37.9			
	Food product	Minerals	0%	2.5%	5%	7.5%			Reference
Cricket	Meat	Iron	0.81 ± 0.16	0.82 ± 0.06	0.94 ± 0.02	1.05 ± 0.01			Cavalheiro et al.,2023
		Zinc	1.22 ± 0.05	1.74 ± 0.03	2.37 ± 0.01	2.66 ± 0.05			
		Potassium	261.80 ± 3.77	258.14 ± 2.81	295.67 ± 1.74	298.51 ± 15.85			
	Food product	Minerals	0%	5%	10%	15%	20%	25%	Reference
Cricket	Biscuits	Iron	13.52 ± 0.96	18.27 ± 0.85	26.19 ± 0.27	31.55 ± 0.45	36.85 ± 0.67	43.33 ± 0.93	Niaba Koffi et al., 2013
		Zinc	3.62 ± 0.12	5.41 ± 0.24	7.36 ± 0.36	7.67 ± 0.48	8.6 ± 0.51	12.85 ± 0.74	
		Phosphorus	250.55 ± 3.21	275.42 ± 2.66	298.52 ± 2.52	365.13 ± 2.89	437.55 ± 2.4	454.68 ± 3.45	
		Potassium	271.17 ± 2.36	340.33 ± 1.25	381.08 ± 3.21	407.33 ± 2.45	453.61 ± 2.7	478.33 ± 3.12	
Edible insects	Food product	Minerals	0%	5%	10%	20%			Reference
Cricket	Biscuits	Sodium	99.7 ± 10.33	103.5 ± 10.29	116.5 ± 10.02	119.2 ± 8.72			Arama, 2025
		Iron	1.3 ± 0.07	1.4 ± 0.02	1.4 ± 0.04	1.5 ± 0.08			
		Manganese	13.2 ± 0.91	13.6 ± 1.19	14.0 ± 0.75	14.5 ± 0.79			
		Zinc	4.2 ± 0.28	7.2 ± 0.75	11.6 ± 4.07	16.8 ± 2.39			
Edible insects	Food product	Minerals	0%	5%					Reference
Cricket	Crackers	Iron	11.29 ± 0.02	12.38 ± 0.05					Ivanišová et al., 2023
		Zinc	11.62 ± 0.08	14.03 ± 0.08					
Edible insects	Food product	Minerals	0%	5%	10%	15%			Reference
Termites	Biscuits	Iron	2.5 ± 0.6	28.5 ± 0.4	34.2 ± 0.5	37.4 ± 0.4			Awobusuyi et al., 2020
		Zinc	2.5 ± 0.5	8.4 ± 0.6	10.4 ± 0.4	14.8 ± 0.4			
		Phosphorus	0.8 ± 0.5	22.5 ± 0.5	31.2 ± 0.4	37.6 ± 0.5			
		Potassium	1.8 ± 0.6	12.5 ± 0.4	18.6 ± 0.5	22.9 ± 0.4			
	Food product	Minerals	0%	5%					Reference
Termite	Bread	Iron	1.20 ± 0.10	1.80 ± 0.22					Kinyuru et al., 2009
		Zinc	2.78 ± 0.60	3.23 ± 0.29					

Edible insects	Food Product	Minerals	40%		References
	Biscuit	Zinc	9.31 ± 0.02		Akande et al., 2020
		Phosphorus	87.20 ± 0.03		
		Potassium	121.80 ± 0.17		
Edible insect	Food product	Minerals	40%		Reference
Locust	Biscuits	Zinc	11.37 ± 0.06		Akande et al., 2020
		Phosphorus	127.70 ± 0.03		
		Potassium	168.22 ± 0.04		
Edible insect	Food product	Minerals			
Palm weevil	Cookies	Iron	109.63 ± 7.00	537.90 ± 30.95	Ogidi et al., 2025
		Zinc	205.00 ± 9.04	321.77 ± 14.88	
		Potassium	217.44 ± 0.04	304.11 ± 21.80	
Edible insect	Food product	Minerals			Reference
	Porridge	Iron	9.86 ± 2.08	19.48 ± 6.69	Maiyo et al., 2022
		Zinc	1.86 ± 0.04	3.71 ± 0.18	
		Phosphorus	221.63 ± 5.57	469.28 ± 9.55	
Edible insect	Food product	Minerals			Reference
	Porridge	Iron	9.86 ± 2.08	9.18 ± 1.18	Maiyo et al., 2022
		Zinc	1.86 ± 0.04	3.39 ± 0.31	
		Phosphorus	221.63 ± 5.57	458.70 ± 3.76	

(*Continued*)

Continued.

Edible insect	Food product	Minerals	0%	10%	Reference
	Snacks	Iron<br>Zinc<br>Phosphorus	32.5<br>103.9<br>36.7	135.0<br>363.6<br>143.3	Roncolini et al., 2020

Edible insect	Food product	Minerals	0%	5%	10%	15%	20%	Reference
Gonimbrasia zambesina	Muffin	Iron<br>Zinc<br>Phosphorus<br>Potassium	4.49±0.19<br>2.31±0.01<br>172.93±3.00<br>156.10±2.40	4.67±0.31<br>2.90±0.06<br>174.7±1.06<br>161.59±5.16	4.79±0.04<br>3.37±0.40<br>177.91±3.80<br>197.36±4.35	4.91±0.04<br>3.37±0.40<br>182.28±4.51<br>242.11±4.32	4.98±0.27<br>3.40±0.14<br>194.19±12<br>273.4±9.25	Ouma et al., 2022

Edible insect	Food product	Minerals	0%	5%	7%	10%	Reference
Grasshopper	Biscuits	Iron<br>Zinc	0.28±0.01<br>0.075	0.40±0.06<br>0.0810	0.36±0.03<br>0.084	0.41±0.11<br>0.109	Dewi et al., 2020

Edible insect	Food product	Minerals	0%	5%	Reference
Grasshopper	Crackers	Iron<br>Zinc	11.29±0.02<br>11.62±0.08	10.08±0.11<br>13.15±0.07	Ivanišová et al., 2023

Edible insect	Food product	Minerals	0%	5%	Reference
Mealworm	Crackers	Iron<br>Zinc	11.29±0.02<br>11.62±0.08	10.91±0.01<br>12.71±0.04	Ivanišová et al., 2023
